# Effects of Reduced Dietary Protein at High Temperature in Summer on Growth Performance and Carcass Quality of Finishing Pigs

**DOI:** 10.3390/ani12050599

**Published:** 2022-02-28

**Authors:** Wenhui Wang, Yifan Chen, Ji Wang, Zhiqian Lv, Enkai Li, Jinbiao Zhao, Ling Liu, Fenglai Wang, Hu Liu

**Affiliations:** State Key Laboratory of Animal Nutrition, College of Animal Science and Technology, China Agricultural University, Beijing 100193, China; wangwh1025@cau.edu.cn (W.W.); chenyfchn@163.com (Y.C.); wang.10819@osu.edu (J.W.); lzq2434390936@163.com (Z.L.); lienkai1994@gmail.com (E.L.); jinbiaozhao@cau.edu.cn (J.Z.); lingliu321@126.com (L.L.); wangfl@cau.edu.cn (F.W.)

**Keywords:** carcass, dietary protein, finishing pigs, heat stress, performance

## Abstract

**Simple Summary:**

It has been well documented that the energy intake and associated energy retention of pigs will decline as the result of heat stress when the ambient temperature increases beyond thermoneutrality. The development of nutritional interventions to alleviate the negative effects of heat stress could be beneficial to pork producers. Studies have shown that reduced protein diets decrease the energy loss related to the excretion of excess N in urine and the energy loss associated with heat production. In conclusion, we provided a new nutritional strategy of decreasing dietary crude protein percentage from 12% to 10% in finishing pigs in summer, which had no negative effects on growth performance and carcass quality.

**Abstract:**

The objective of this experiment was to investigate the effects of reduced dietary protein at natural high temperature in summer on the growth performance and carcass quality of finishing pigs. A total of 72 crossbreed pigs (Duroc × Landrace × Yorkshire) at an average body weight (BW) of 77 ± 5.7 kg were randomly assigned to two treatments, based on BW and sex, in six replicates per treatment, with six pigs per pen, using a randomized complete block design. The dietary crude protein (CP) level of the normal protein diet (NP) and the reduced protein diet (LP) were 12% and 10%, respectively. The growth performance and serum biochemical parameters of the pigs were analyzed for a 28-day experimental period. At the end of the experiment, 12 pigs were harvested to measure carcass characteristics and pork quality. The average highest ambient temperature during the experiment period was about 32.4 °C. There was a trend for the average daily feed intake (ADFI) to be lower in the pigs on the reduced protein diet compared to the control (*p* < 0.10) in the 0–28 day period. The serum urea nitrogen was lower (*p* < 0.05) for pigs fed the reduced protein diets only on day 14. The carcass characteristics and pork quality were not affected by dietary treatments. In conclusion, decreasing dietary crude protein percentage from 12% to 10% in finishing pigs in summer may have no negative effects on growth performance and carcass quality.

## 1. Introduction

When fattened pigs are managed under thermoneutral conditions, dietary energy is used to meet their requirements for maintenance, physical activity, and growth [1]. If the ambient temperature increases beyond thermoneutrality, the energy intake and associated heat loss and energy retention (ER) of pigs will decline as a result of heat stress (HS) [2]. In hotter climates, livestock production is severely affected by HS [3]; with economic losses originating from increased morbidity, mortality, suboptimal growth, inefficient nutrient use, reduced muscle mass, and increased adipose tissue deposition [4]. Development of nutritional interventions to alleviate the negative effects of HS could be beneficial to pork producers in hotter climates.

The nutrient strategy of a dietary decrease in protein percentage by 2% to 4%, supplemented with limiting amino acids (AA), for finishing pigs has two primary benefits [5]. It helps to decrease the amount of fecal nitrogen excretion to the environment, without affecting growth performance [6,7,8]. Additionally, feeding reduced protein diets to pigs decreases the energy loss related to the excretion of excess N in urine and the energy loss associated with heat production [9]. However, the effects of the reduction of dietary crude protein (CP) levels in finishing pigs under natural high temperatures in summer on growth and carcass quality is unclear. Hence, we hypothesized that decreasing dietary crude protein percentage in finishing pigs in summer could alleviate heat stress and have no negative effects on growth performance and carcass quality.

Therefore, the objective of this study was to determine the effects of reducing the percentage of dietary protein in diets fed to finishing pigs at natural high temperature in summer on growth performance and carcass quality.

## 2. Materials and Methods

### 2.1. Animals, Diets, and Experimental Design

A total of 72 crossbred pigs (Duroc × Landrace × Yorkshire) with an average initial body weight (BW) of 77 ± 5.7 kg were blocked by weight and sex. Pigs were randomly allotted to two treatments, and each dietary group had 6 replicates per pen (3 barrows and 3 gilts). The experiment was carried out at the Swine Nutrition Research Centre (Zhuozhou, Hebei Province, China; 39°28′ N, 115°51′ E). The pens were 2.4 × 2.8 m group-feeding pens with partially slatted concrete floors. Each pen was equipped with a one-sided self-feeder and a nipple waterer to allow the pigs ad libitum access to feed and water.

Two experimental diets (Table 1) based on corn, soybean meal, and wheat bran were prepared. A conventional protein diet was the control diet and was formulated to meet or exceed NRC (2012) recommendations for pigs in that BW range [10]. In the low protein diet, crude protein (CP) percentage was reduced by substitution of soybean meal with corn, wheat bran, and soybean oil. The calculations were based on the net energy (NE) values of feedstuffs [11] and formulated to obtain similar NE values for the two diets. These values met NRC (2012) recommendations [10].

The experiment was conducted under natural diurnal high temperature natural conditions, without air conditioning. The ambient temperature and relative humidity inside the barn were recorded during the whole study with a temperature and humidity data logger (RC-4HA/C; Elitech Technology, Inc. Xuzhou, Jiangsu Province, China). The logger recorded values every 6 min, which were used to calculate the humidex [12]. Rectal temperatures and respiratory frequency were measured at 13:00 on the last 3 days of the experiment and averaged [2].

Pigs were weighed individually on days 0, 14, and 28, to determine average daily gain (ADG). The feed consumption of each pen during days 0–14 and days 15–28 were recorded to calculate the average daily feed intake (ADFI). Gain:feed (G:F) was then calculated from ADG and ADFI.

### 2.2. Blood Collection and Animal Sampling

Blood samples were collected from one pig per pen via the anterior vena cava into 10-mL tubes containing no anticoagulant (Becton Dickinson Vacutainer Systems, Franklin Lakes, NJ, USA) on days 0, 14, and 28 following an overnight fast. Samples were centrifuged (Biofuge22R; Heraeus, Hanau, Germany) at 3000× *g* for 10 min, and the serum was kept at −80 °C until analyzed.

At the end of the experiment, 6 pigs (3 barrows and 3 gilts) whose final BW was closest to the average BW of the respective pen (one pig per pen) were randomly selected. A total of 12 pigs were transported and harvested at the Laboratory of Animal Metabolism of China Agricultural University (Beijing, China) after an overnight fast. Pigs were slaughtered in an abattoir by exsanguination after electrical stunning. Dressing percentage, carcass length, longissimus dorsi muscle area (LMA), fat depth, as well as 45-min and 24-h pH was determined, according to reported methods [8]. Carcass fat-free lean gain was calculated according to the equation of NPPC [13].

Meat loin samples from the m. longissimus were taken near the last rib to measure meat quality traits, such as drip loss color and shear force. Meat color, including lightness (L*), redness (a*), and yellowness (b*) values, was measured by a colorimeter (Chroma meter CR-410; Minolta, Tokyo, Japan). Drip loss for 24 h was determined by using a reported method [14]. Loin sections of about 100 g weight and 3-cm thickness were placed in plastic bags and cooked to an internal temperature of 70 °C on a thermostat water bath. Then, 10 cylindrical samples (approximately 10 mm diameter × 10 mm length) were cut from the center of the muscle with their axis parallel to fiber direction. The shear force of the cooked meat was determined by using a digital-display muscle tenderness meter (C-LM3B, Tenovo, Harbin, China).

### 2.3. Chemical Analysis

Duplicate ingredient and diet samples were analyzed according to the AOAC (2007) procedure [15], including dry matter, crude protein, total phosphorus, calcium, and ether extract [16]. Neutral detergent fiber and acid detergent fiber were determined using filter bags and fiber analyzer equipment (Fibre Analyzer; Ankom Technology, Macedon, NY, USA), following a modified procedure [17]. Analysis of amino acid (AA) content included in the ingredients and diets was conducted according to a reported method [18].

After the frozen serum samples were thawed at 4 °C, serum levels of glucose, total protein, albumin, globin, triglyceride, total cholesterol, and urea nitrogen were quantified using a biological analyzer (7600 Automatic Biological Analyzer; Hitachi, Tokyo, Japan) at the Kangjia Hongyuan Biotech Company (Beijing, China). Serum heat stress protein 70 (HSP 70) concentration was measured using a microplate reader (Multiskan MK3 Microplate Reader, Waltham, MA, USA) at the Kangjia Hongyuan Biotech Company (Beijing, China).

### 2.4. Statistical Analysis

All data were analyzed using the general linear model (GLM) procedures of SAS 9.2 (SAS Institute Inc., Cary, NC, USA). The linear model included terms for treatment (fixed), block (fixed), and treatment × block (fixed). Pens were considered the experimental unit for growth performance analysis, the individual pigs served as the experimental unit for other indexes. Probability levels of *p* < 0.05 and 0.05 < *p* ≤ 0.10 were defined as significant differences and tendencies, respectively.

## 3. Results

### 3.1. Humidex, Rectal Temperature and Respiratory Frequency

**Figure 1:**
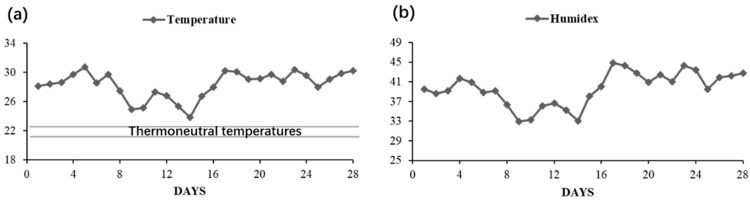

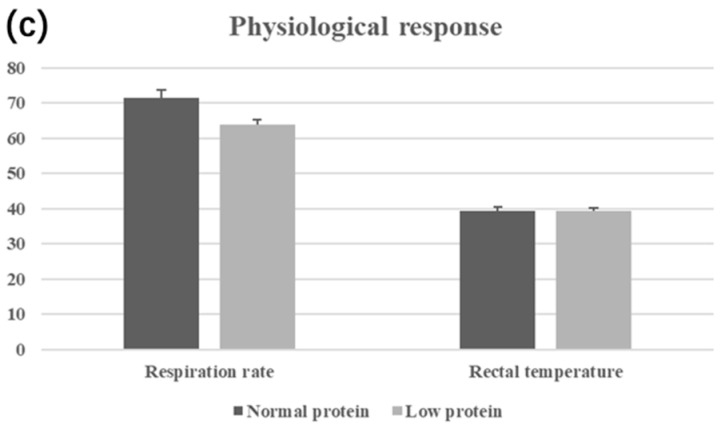
Environmental parameters and physiological response of finishing pigs during trial period. (**a**) Average full-day trial period barn temperature; (**b**) average full-day trial period barn humidex; (**c**) respiration rate (breaths/min) and rectal temperature (°C).

The average daily experimental period humidex is shown in Figure 1. The humidex values were between 33 to 45. Rectal temperatures (39.3 °C vs. 39.4 °C) and respiratory frequency (72 breaths/min vs. 64 breaths/min) were not significantly different between treatments (*p* > 0.05).

### 3.2. Performance

The effects of dietary crude protein level on the growth performance of the finishing pigs at high temperature in summer is presented in Table 2. Reduced dietary protein did not influence ADG, ADFI, or F:G in the 0–14 day and 15–28 day phases (*p* > 0.05). However, during the overall experimental period there was a slight decrease of ADFI in pigs fed the low protein diet (*p* < 0.10).

### 3.3. Carcass Characteristics and Pork Quality

As shown in Table 3, the results of carcass analysis showed no significant differences between treatments in carcass weight, dressing percentage, carcass length, 10th rib fat depth, LMA, or fat-free lean (*p* > 0.10).

The effects of reduced dietary protein level on meat quality at high temperature in summer are provided in Table 4. Minolta scores (L*, a*, and b* value), pH_45min_, pH_24h_, drop loss, and shear force were not significantly influenced by reduced dietary protein (*p* > 0.10).

### 3.4. Serum Biochemical Parameters

The effect of dietary crude protein level on the serum biochemical parameters of finishing pigs at high temperature in summer is presented in Table 5. Reduced dietary protein did not influence serum biochemical parameters on days 0 and 28 (*p* > 0.10). However, pigs fed a reduced protein diet had a lesser serum urea nitrogen concentration on day 14 of the trial (*p* < 0.05).

## 4. Discussion

### 4.1. Humidex, Rectal Temperature, and Respiratory Frequency

Humidex was developed for the Meteorological Service of Canada and describes the ‘feels-like’ or heat index temperature for humans and livestock [12]. The index has a series of thresholds, i.e., 30 indicates some discomfort, 46 indicates dangerous heat index, and 54 indicates a heat index likely to result in imminent heat stroke [19]. The humidex of the barn during the experiment period was higher than 30, which indicates that the pigs suffered heat stress during the 28-day experiment. The physiological responses to ambient temperature, such as rectal temperatures and respiratory frequency were not significantly different between treatments. When compared with the previous studies related to heat stress, the respiratory frequency (72 and 64 breaths/min) was able to show that the pigs in this experiment suffered heat stress [20].

### 4.2. Performance

It has been well documented that HS decreases the growth performance of pigs, i.e., a reduction in ADFI and ADG [3,5]. The current experiment did not include treatments under thermoneutral conditions as controls, but the ADFI, ADG, and F:G in this experiment clear reflected the higher summer heat indexes compared to results from studies conducted in thermoneutral conditions [21]. Considering how HS detrimentally affects energetic homeostasis and nutrient partitioning in pigs may allow for future nutritional interventions to mitigate the harmful effects of increased environmental temperature [3]. Reducing feed intake is suggested as a strategy to minimize metabolic heat production, as is decreasing physical activity [5,22]. Additionally, adipose tissue mobilization under short-term HS and skeletal muscle catabolism during chronic HS in pigs may partially contribute to acute loss of BW [3].

A reduced protein diet was designed in the present research to determine the effects of dietary protein reduction on performance under high temperature conditions in summer. Our data indicated that the reduced dietary protein at natural high temperature in summer had no effect on growth performance from days 0–14 and days 15–28, which concurs with previous reports [6,7,23]. ADFI was only less favorable in the pigs on a low protein diet during the day 0–28 period. There was no difference in F:G, which indicated that dietary protein level had no negative effect on feed conversion efficiency. It is important to note that the diets used in the current study were formulated to contain equal amounts of standardized ileal digestible concentrations of Lys, Met, Thr, and Trp, according to NRC (2012) requirements [7,24]. This could be interpreted, that reducing the CP content in the finishing diets from 12.2 to 10.0% with adequate supplementation of limiting amino acids according to the ideal protein concept does not affect growth performance [25].

### 4.3. Carcass Characteristics and Pork Quality

In the current research, pigs fed 10% CP diet supplemented with crystalline AA had similar carcass characteristics to pigs fed 12% CP diet, which is similar to reports in the literature [23,25,26,27]. It is possible that the effects of reduced dietary protein on carcass traits may mitigate by supplementation with the proper AA [28]. However, this was different from the findings of [29], who found that pigs fed a reduced protein diet supplemented with AA produced carcasses with slightly less lean mass than pigs fed a control diet. A cyclical ambient temperature during the day and night in summer was recorded, which indicated that pigs suffered lesser heat stress compared with the consistent HS regimen. This may explain the above difference in outcomes, where the approximate dietary protein level resulted in differences in carcass traits.

In the present study, the effect of reduced protein diet at high natural temperature in summer on the meat quality of finishing pigs was also measured. Our data indicated that meat quality, including meat color, drip loss, shear force, and pH_45min_ and pH_24h_ were similar for the two treatments, consistent with other results in the literature [30]. These results were also in agreement with the reports that dietary protein level had no significant effect on pork meat quality [31,32]. In research evaluating the effects of ambient temperature, there were no interactions between dietary lysine level and environmental temperature on pork quality [33].

### 4.4. Serum Biochemical Parameters

One of the main nutritional benefits of reduced protein diets for pigs is to improve the efficiency of nitrogen utilization, with reduced nitrogen breakdown and excretion [5,34]. The serum urea nitrogen (SUN) concentration can be an indirect indicator of the AA utilization in the diet, as declines in SUN reflect an improved efficiency of nitrogen utilization and lesser synthesis of urea [35,36,37]. In our research, the SUN concentrations of pigs fed a reduced protein diet was significantly lower than in pigs fed a control diet on day 14, which was consistent with the results of [38,39]. This may be due to improved nitrogen utilization efficiency [23]. Serum biochemical parameters, such as glucose, triglycerides, and total cholesterol were similar. These results indicated that reduced dietary protein at high temperature in summer had little effect on glycolipid metabolism, which was consist with previous studies [40,41]. HSP70 is a universal cytoprotective protein which enhances tolerance to environmental changes and, therefore, increases the survival rate of stressed cells [42,43]. Heat stress is a potential causative factor in activating and upregulating heat shock proteins [3]. In the present experiment, the serum HSP70 concentration were similar between treatments. Considering all pigs in the current experiment were under natural diurnal high temperature conditions, the results of serum HSP70 concentration at least indicated that the reduced protein diets at high temperature in summer did not cause the extra HSP70 release.

## 5. Conclusions

In conclusion, reduced dietary protein, from 12% to 10%, at natural high temperature in summer had no effect on performance, carcass characteristics, and pork quality in finishing pigs.

## Figures and Tables

**Table 1:** Ingredient composition of the experimental diets (%, as-fed basis).

Item	Crude Protein Level ^1^	
	NP	LP
Ingredients		
Corn	76.25	81.00
Soybean meal	7.96	0.00
Wheat bran	12.82	15.32
Soybean oil	0.00	0.13
Dicalcium phosphate	0.45	0.50
Limestone	0.98	1.01
Salt	0.35	0.35
Vitamin and mineral premix ^2^	0.50	0.50
L-Lysine HCL	0.44	0.67
DL-Methionine	0.04	0.07
L-Threonine	0.13	0.23
L-Tryptophan	0.03	0.07
L-Valine	0.05	0.15
Total	100.00	100.00
Analyzed levels		
Dry matter	88.60	88.53
Crude protein	12.23	9.98
Ether extract	2.23	2.55
Neutral detergent fibre	15.13	15.26
Acid detergent fibre	3.71	3.41
Calcium	0.53	0.52
Total phosphorus	0.48	0.47
NE, kcal/kg ^3^	2476.10	2476.10
SID amino acids ^4^		
Lys	0.73	0.73
Met	0.22	0.21
Thr	0.46	0.46
Trp	0.13	0.13
Val	0.48	0.48

^1^ NP, normal protein diet (12% CP); LP, low protein diet (10% CP). ^2^ Supplied the following amounts of vitamin-mineral per kilogram of diet: 5512 IU vitamin A, 30 IU vitamin E, 2200 IU vitamin D3, 27.6 μg vitamin B12, 2.2 mg vitamin K3, 14 mg pantothenic acid, 4 mg riboflavin, 30 mg niacin, 0.7 mg folic acid, 400 mg choline chloride, 3 mg pyridoxine, 1.5 mg thiamine, 44 μg biotin, 75 mg Fe (FeSO_4_·H_2_O), 40 mg Mn (MnO), 100 mg Cu (CuSO_4_·5H_2_O), 75 mg Zn (ZnO), 0.3 mg Se (Na_2_SeO_3_), and 0.3 mg I (KI). ^3^ NE content of the diets was calculated using energy values for the ingredients obtained from [11]. ^4^ The standardized ileal digestible (SID) concentrations were determined by multiplying the determined AA content in ingredients by the SID coefficients of the corresponding AA in those feedstuffs from NRC (2012) and summing the values.

**Table 2:** Effects of dietary crude protein level on the growth performance of finishing pigs ^1^.

Item	Crude Protein Level ^2^		SEM	*p* Value
	NP	LP		
Initial BW, kg	76.3	78.8	2.1	0.398
Final BW, kg	93.4	94.5	1.9	0.274
0–14 days				
Average daily feed intake, kg	2.34	2.23	0.07	0.275
Average daily gain, kg	0.55	0.47	0.64	0.358
Feed:gain	4.33	5.14	0.50	0.269
15–28 days				
Average daily feed intake, kg	2.48	2.25	0.11	0.182
Average daily gain, kg	0.67	0.65	0.40	0.672
Feed:gain	3.73	3.48	0.22	0.432
0–28 days				
Average daily feed intake, kg	2.41	2.24	0.05	0.054
Average daily gain, kg	0.61	0.56	0.03	0.239
Feed:gain	3.98	4.08	0.26	0.766

^1^ Data are means of 6 replicates per treatment with 6 pigs per pen. ^2^ NP, normal protein diet (12% CP); LP, low protein diet (10% CP); SEM, standard error of mean.

**Table 3:** Ingredient composition of the experimental diets (%, as-fed basis) ^1^.

Item	Crude Protein Level ^2^		SEM	*p* Value
	NP	LP		
Slaughter weight, kg	95.2	92.4	2.9	0.520
Carcass weight, kg	72.9	69.3	2.7	0.370
Dressing percentage, %	76.57	74.92	1.06	0.296
Carcass length, cm	92.12	91.75	1.31	0.847
The tenth rib fat depth, cm	2.17	2.03	0.13	0.492
Longissimus muscle area, cm²	37.95	35.74	2.84	0.595
Fat-free lean, %	51.46	51.75	1.55	0.896

^1^ Data are the means of 6 replicates per treatment with 6 pigs per pen. ^2^ NP, normal protein diet (12% CP); LP, low protein diet (10% CP).

**Table 4:** Effects of dietary crude protein level on the meat quality of finishing pigs ^1^.

Item	Crude Protein Level ^2^		SEM	*p* Value
	NP	LP		
Meat color ^3^				
L*	47.48	49.86	1.77	0.365
a*	15.88	16.33	0.57	0.585
b*	1.41	1.91	0.45	0.452
Drip loss, %	1.83	2.67	0.73	0.434
Shear force, N	48.27	45.25	3.41	0.545
pH				
pH_45min_	5.58	5.81	0.23	0.487
pH_24h_	5.37	5.33	0.04	0.5

^1^ Data are the means of 6 replicates per treatment with 6 pigs per pen. ^2^ NP, normal protein diet (12% CP); LP, low protein diet (10% CP); ^3^ L*, lightness; a*, redness; b*, yellowness.

**Table 5:** Effects of dietary crude protein level on the serum biochemical parameters of finishing pigs ^1^.

Item	Crude Protein Level ^2^		SEM	*p* Value
	NP	LP		
0 days				
Glucose, mmol/L	3.82	3.87	0.38	0.932
Urea nitrogen, mmol/L	3.36	2.52	0.42	0.187
Total protein, g/L	63.98	65.52	3.63	0.772
Albumin, g/L	27.22	25.57	1.48	0.449
Globin, g/L	36.77	39.96	3.24	0.503
Triglyceride, mmol/L	0.40	0.45	0.05	0.504
Total cholesterol, mmol/L	1.94	2.09	0.17	0.571
Heat stress protein 70, pg/mL	143.48	147.94	4.41	0.490
14 days				
Glucose, mmol/L	4.27	4.50	0.42	0.701
Urea nitrogen, mmol/L	3.63	2.16	0.34	0.013
Total protein, g/L	71.22	67.25	2.74	0.331
Albumin, g/L	29.68	27.25	1.34	0.229
Globin, g/L	41.53	40.00	2.66	0.692
Triglyceride, mmol/L	0.41	0.43	0.05	0.729
Total cholesterol, mmol/L	2.15	2.18	0.13	0.859
Heat stress protein 70, pg/mL	159.69	164.15	8.45	0.717
28 days				
Glucose, mmol/L	3.69	4.25	0.32	0.241
Urea nitrogen, mmol/L	3.80	3.38	0.31	0.382
Total protein, g/L	52.79	58.33	5.40	0.484
Albumin, g/L	24.05	26.50	2.17	0.444
Globin, g/L	28.73	31.83	4.06	0.601
Triglyceride, mmol/L	0.38	0.48	0.09	0.467
Total cholesterol, mmol/L	1.68	1.92	0.21	0.438
Heat stress protein 70, pg/mL	141.55	139.65	5.08	0.796

^1^ Data are the means of 6 replicates per treatment with 6 pigs per pen. ^2^ NP, normal protein diet (12% CP); LP, low protein diet (10% CP).

## Data Availability

Some or all data, models, or code generated or used during the study are available in a repository or online in accordance with funder data retention policies (Provide full citations that include URLs or DOIs.).
